# Complete sequence of mitochondrial DNA of *Gracilaria tenuistipitata* (Rhodophyta)

**DOI:** 10.1080/23802359.2018.1464410

**Published:** 2018-07-25

**Authors:** Na Liu, Yue Li, Cui Liu, Tao Liu, Weizhou Chen

**Affiliations:** a College of Marine Life Sciences, Ocean University of China, Qingdao, China;; b Marine Biology Institute, Shantou University, Shantou, China

**Keywords:** *Gracilaria tenuistipitata*, mitogenome, phylogenetic tree

## Abstract

We sequenced and analysed the complete mitogenome of *Gracilaria tenuistipitata*. The complete *G. tenuistipitata* mitogenome is 25,819 bp long, containing 50 genes, including 24 protein-coding genes, one intron, two rRNA genes, 23 tRNA genes, and one unidentified open reading frame. Twenty-three of the 24 (95.83%) protein-coding genes ended with the TAA stop codon, and one (4.17%) with TAG (*rps*3 gene). All protein-coding genes in *G. tenuistipitata* were found to contain the start codon ATG. Phylogenetic analysis revealed that *G. tenuistipitata* clustered with *G. chilensis.* The complete mitochondrial genome sequence provided here should be useful for elucidation of *Gracilaria* evolution.

In Taiwan, *Gracilaria* species have been cultivated since 1961 (Ajisaka and Chiang 1993). The major *Gracilaria* species produced by open sea cultivation are *G. tenuistipitata*, *G. confervoides*, *G. gigas*, *G. chorda*, and *G. compressa*. Bioactivities of marine algae of the genus *Gracilaria* have been extensively studied (de Almeida et al. 2011). Aqueous extracts of *G. tenuistipitata* have an antioxidant activity and protective effect against H_2_O_2_-induced DNA damage and can inhibit hepatitis C virus 9 (HCV) replication at nontoxic concentrations (Yang et al. 2012). In addition, ethanolic and methanolic extracts of *G. tenuistipitata* exert antiproliferative action on (and induce apoptosis in) oral cancer cells (Yeh, Tseng, et al. 2012; Yeh, Yang, et al. 2012). Until now, however, no genomic studies on *G. tenuistipitata* have been reported.

Herein, we determined the complete *G. tenuistipitata* mitogenome sequence. Genomic DNA from one *G. tenuistipitata* individual collected from a population in China (21°41′2″N, 108°21′4″E) was used for sequencing. The specimen (accession number: 2016040159) was deposited in the Culture Collection of Seaweeds at the Ocean University of China. Paired-end sequencing reads were obtained on an Illumina HiSeq × Ten system (Illumina, ‎San Diego, CA). Approximately, 9 Gb of paired-end (125 bp) sequence data was randomly extracted from the total sequencing output, as input to NOVOPlasty (Dierckxsens et al. 2017) for assembling the mitogenome. *Gracilaria salicornia* (GenBank accession number: NC_023784) served as the seed sequence. tRNA genes were identified on the tRNAscan-SE Search Server (Schattner et al. 2005). Other mitogenomic regions were annotated by means of the *G. salicornia* mitogenome via Geneious R10 (Biomatters Ltd., Auckland, New Zealand). Phylogenetic mitogenome analysis was conducted. Bayesian inference was performed in the MrBayes software v.3.1.2 (Huelsenbeck and Ronquist 2001). The phylogenetic analysis was conducted in two independent runs with four Monte-Carlo Markov Chains running for 1,000,000 generations. Output trees were sampled every 100 generations. The phylogenetic analysis was run until the average standard deviation of split frequencies decreased below 0.01 and the first 25% of samples was removed as burn-in. *Rhodymenia pseudopalmata* (KC875852) served as the outgroup.

The complete *G. tenuistipitata* (MG592727) mitogenome represents a circular DNA molecule measuring 25,819 bp in length. Overall A + T content of the complete mitogenome is 72.9%. The mitogenome contains 50 genes, including 24 protein-coding, two rRNA, and 23 tRNA genes and one unidentified open reading frame. Twenty-three of the 24 (95.83%) protein-coding genes end with the TAA stop codon, and one (4.17%) with TAG (*rps*3 gene). All protein-coding genes in *G. tenuistipitata* were found to contain the start codon ATG. The lengths of two rRNA genes are 2624 bp (*rnl* rRNA) and 1401 bp (*rns* rRNA). Bayesian inference showed that *G. tenuistipitata* clustered together with *G. chilensis* (Figure 1). The complete mitogenome sequence provided herein should help understand *Gracilaria* evolution.

**Figure 1.**
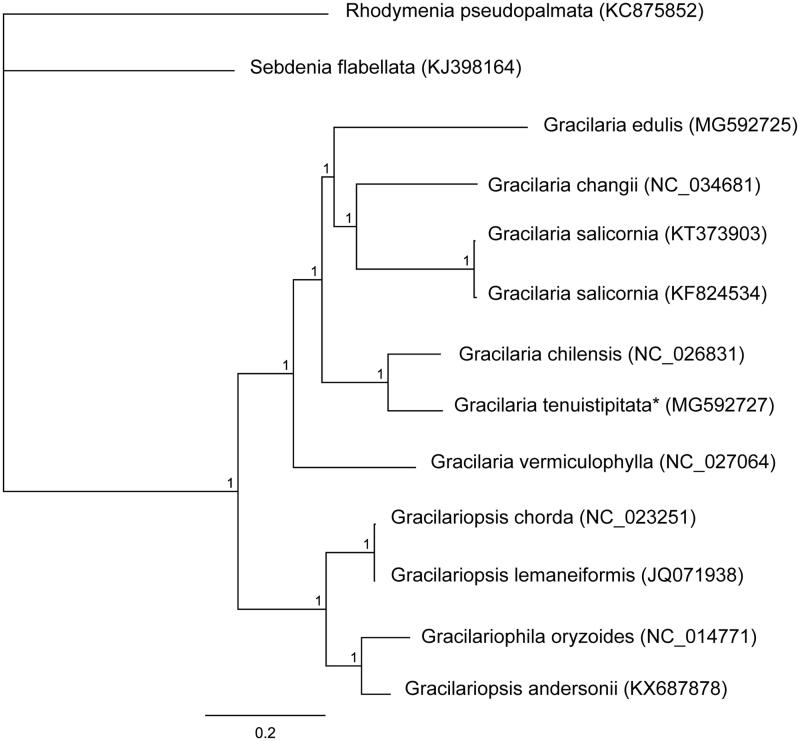
Phylogenetic tree (Bayesian inference) based on complete mitogenomes of species within Gracilariaceae. Support values for each node were calculated from Bayesian posterior probability (BPP). Asterisks following species names indicate newly determined mitogenomes.
